# Intercultural Scale on Conceptions of Disability: a cross-cultural validation

**DOI:** 10.1186/s41155-026-00379-0

**Published:** 2026-03-14

**Authors:** Lúcia Pereira Leite, Gisele Magarotto Machado

**Affiliations:** 1 Department of Psychology and the Postgraduate Program in Developmental and Learning Psychology, São Paulo State University (UNESP), Av. Eng. Luiz Edmundo Carrijo Coube, 14-01 - Vargem Limpa, Bauru/SP, 17033-360 Brazil; 2 Division of Mental Health Services, R&D Department, Akershus University Hospital, Lørenskog, Norway; 3 Department of Psychology, PROMENTA Research Center, University of Oslo, Oslo, Norway

**Keywords:** Disability, Scale, Cross-cultural validation, Conception

## Abstract

**Objective:**

Our study investigates the cross-cultural validation of the Intercultural Scale on Conceptions of Disability (ISCD), which measures how disability is perceived across three dimensions: biological, social, and metaphysical.

**Methods:**

The research involved 5,544 undergraduate and graduate students from Brazil, Portugal, and Spain. Confirmatory factor analysis (CFA) was used to evaluate the scale’s three-factor structure in each country. Measurement invariance was also tested to ensure that the scale measures conceptions of disability equivalently across all cultural contexts.

**Results:**

The dates showed that the ISCD's three-factor structure demonstrated a good fit in all locations, confirming the multidimensional nature of disability conceptions. Additionally, measurement invariance was established, indicating that the instrument's parameters were consistent across countries. Significant differences were found between countries in all three conceptions. The study also provides normative data for interpreting the ISCD scores, which is crucial for future research and applications in diverse contexts.

**Conclusion:**

The findings suggest that the ISCD is a valid instrument for assessing disability conceptions across different cultures, offering valuable insights for developing inclusive policies and interventions that consider the social and cultural dimensions of disability.

**Supplementary Information:**

The online version contains supplementary material available at 10.1186/s41155-026-00379-0.

## Introduction

Conceptually, there are different ways to conceive and interpret disability, which translate into varied behaviors and attitudes of society towards individuals in this condition (Gallagher et al., 2014; Pereira-Leite et al., 2023a, 2023b). The discussion surrounding the recognition of people with disabilities as rights-bearing individuals is relatively recent. In this context, recognizing people with disabilities as subjects of rights and understanding them from a perspective that values ​​human diversity has become the focus of action. This is particularly evident in the promulgation of the United Nations Convention on the Rights of Persons with Disabilities – CRPD (UN, 2006), an international treaty that codifies the rights of people with disabilities to ensure their participation in various social spheres.

The shifts in both the conception of disability and the treatment of individuals with disabilities became more pronounced following the adoption of the Convention on the Rights of Persons with Disabilities (United Nations, 2006, p.4). Article 1 of the document defines persons with disabilities as: "those who have long-term physical, mental, intellectual or sensory impairments which in interaction with various barriers may hinder their full and effective participation in society on an equal basis with others." The primary goals of the Convention are to support, advocate for, and ensure that all individuals with disabilities have equal access to human rights and fundamental freedoms, while also promoting respect for their dignity. Consequently, this represents a broader and more social conception of disability, emphasizing the presence of barriers as the main limitations to participation in everyday spheres of life.

As indicated by previous studies (Moreira et al., 2022; Nepomuceno et al., 2020), even with the recent increase in the participation of individuals with disabilities in common social spheres, especially in the workplace and in education, society’s relationship with these individuals remains marked by prejudice. This relationship is still filled with labels that convey notions of inferiority, with terms such as "incapable," "invalid," "inefficient," "abnormal," and "exceptional" being commonly used. These terms are deeply ingrained with the idea of deficiency, implying that these individuals are viewed as second-class citizens (De Menezes, 2018). This reflects the dominance of an organic perspective that characterizes disability, where the standard of a healthy body is one that is whole or complete. The organic perspective, still prevalent in contemporary society, aligns with the biomedical model of disability, which conceptualizes it as an inherent organic attribute of the individual. Within this framework, disability is equated with pathology, whereby individuals are classified as disabled solely based on the diminished function of a body part relative to socially constructed norms of normality. This perspective contributes to stigmatization and typically results in individuals being directed toward medical services for correction or intervention (Jain & Harris, 2024). Both students' and teachers' attitudes toward disability are often shaped by this biological viewpoint, which hinders greater social participation and inclusion (Moriña & Carnerero, 2020).

It is well known that the concept of disability is complex and results from an interaction between the characteristics of the human body (such as sensory, intellectual, motor, and emotional functions) and the characteristics of the society in which individuals live (such as negative attitudes, inaccessible transportation and public buildings, and lack of social support). This interaction can sometimes deprive individuals of their right to fully participate in society (Gairín & Suárez, 2014). Individuals who present different physical or behavioral traits are often subjected to strategies of symbolic violence (Mazzotta & D'Antino, 2011). This idea suggests that many of the limitations faced by people with disabilities are related to the environments in which they live. Additionally, disability can also be understood through a religious lens (Strelhow, 2018). For instance, Christianity influences current interpretations of disability through common discourses that depict individuals with disabilities as either a divine gift, a bad omen, or even as angels or enlightened beings.

Thus, when addressing the concept of disability, it is crucial to relate it to the cultural context and historical period in which it occurs. In the current landscape, there is a notable spread of governmental actions claiming to be inclusive, especially in developed or developing countries^1^ aimed at supporting individuals with disabilities. However, in contrast, this same context, shaped by a socioeconomic model that prioritizes productivity and perfection, distances the concept of disability from what is considered ideal. The classification of these differences as dysfunctions stems from their divergence from the socially constructed and increasingly enforced standard of bodily normativity. As a result, ableism, a form of discrimination directed at people with disabilities (Mello, 2020), becomes entrenched. In this framework, individuals with disabilities are pressured to conform to standards of appearance and functionality associated with the idea of a healthy body, one that acts competently and autonomously in response to societal demands, following a logic of functionality and productivity.

Drawing from the readings of historical-cultural psychology, particularly Vygotsky's work Defectology (1997), it is inferred that disability should be interpreted as a dynamic, relational, and processual state, considering the relationship between the individual and the sociocultural context. In other words, the differentiated individual conditions of human development are shaped by the expectations and attitudes directed at the individual, making disability not just an event but, in our view, a social category of analysis. Revisiting Vygotsky's ideas (2010), it is understood that the way the concept is disseminated and appropriated, whether theoretically or procedurally, shapes how it is conceived and the attitudes associated with it.

In complement, Waldschmidt et al. (2017), who adopts a cultural model of disability, argues that culture is a social practice and an analytical category, grounded in the idea that all objects (material and immaterial), institutions, languages, and behaviors created and used by a social group reverberate in the attitudes and identity formation of its members. In examining the social implications of disability, Barroco and Leite (2021) and Valle and Connor (2014) emphasize that disability is still commonly associated with negative attributes, where bodies that differ from dominant norms are labeled as dysfunctional. This reinforces the idea of being divergent or abnormal, as dictated by cultural standards. According to Piccolo and Mendes (2022), when disability is viewed as an unavoidable tragedy, there emerges a perceived need to create mechanisms to treat or cure it, with health services assuming a central role in attempts to restore or approximate the individual to “normality.” Consequently, stigmas, prejudices, and stereotypes are constructed around disability and those who experience it, which often limit individuals’ autonomy, as they come to bear socially assigned negative labels and are perceived as deviating from “normal” people due to bodily, cognitive, and/or behavioral differences. These factors can lead to individuals with disabilities being seen as lesser subjects, destined to fail or dependent on charitable or assistance-based responses, rather than being recognized as individuals with rights, like any other citizen (Mello, 2020).

Even though disability is a widely discussed concept, a review of scientific databases revealed a scarcity of robust research instruments aimed at identifying conceptions or interpretations of disability. A systematic integrative review (Moraes et al., n.d. in press), which aimed to measure how different population segments understand disability, identified ten articles published between 2014 and 2024 through a search of electronic databases such as Web of Science and Scopus. Different studies have used instruments developed to assess conceptions of disability, designed by researchers from countries such as Italy, Spain, Japan, Canada, the United States, Brazil, and Germany. Among the most relevant are the Chedoke McMaster *Attitudes Toward Children with Handicaps* (CATCH), which assesses children's attitudes toward peers with disabilities (Derguy et al., 2021); the *Disability Social Work* (DSW) scale, which investigates feelings and perceptions about disability (Iwakuma et al., 2023); and the *Attitudes to Disability Scale* (ADS), which examines perceptions about the functioning and participation of people with disabilities in society (Zaluska et al., 2020). In addition to these, the *Escala de Actitudes hacia la Discapacidad* (EAD) (López-Bastías & Moreno-Rodríguez, 2019) and the *Escala de Concepções de Deficiência* (ECD) (Leite & Lacerda, 2018) address different dimensions of how disability is understood. Other instruments include questionnaires focused on teachers' perceptions (Rizzo et al., 2021), a self-assessment tool to identify social attitudes and environmental barriers (Garcia et al., 2015), and the development of intercultural item banks to assess social attitudes toward disability (Upegui-Arango et al., 2022). Despite growing global interest in the topic, there are still a limited number of internationally validated instruments in this area. In this context, it is essential to develop and validate investigative tools (measurement instruments) capable of capturing how individuals and social groups conceptualize disability and refer to those who experience it. In line with this need, the present study aims to validate a measure of conceptions of disability across different cultural contexts.

Given this gap on the literature, a group of researchers from Brazil, Portugal, Spain, and Cuba, specializing in Psychology and Education, sought to develop a research instrument aimed at identifying and assessing conceptions of disability among university students based on three dimensions: metaphysical/religious, biological, and social. This instrument, named the Intercultural Scale on Conceptions of Disability (ISCD) (Leite, 2019), was applied to a sample of Brazilian university students, and the ISCD demonstrated evidence of validity based on its internal structure, including factor structure and internal consistency (Leite et al., 2021). To date, this is the only study that has investigated the psychometric properties of the scale, which highlights the need for further research with larger samples and in different cultural contexts.

Accordingly, the aim of the present study was to investigate the validity of the ISCD in countries beyond Brazil, specifically in Portugal and Spain, while also reaffirming its validity within the Brazilian context, now using a more diverse sample that reflects the country’s continental dimensions. The choice of these countries is justified by the fact that they are signatories of the Convention on the Rights of Persons with Disabilities and therefore should share similar conceptions of disability. Moreover, the researchers who contributed to the development of the ISCD were affiliated with universities in these countries. In addition to evaluating the internal structure of the measure, this study sought to examine the measurement invariance of the instrument across the three countries, in order to verify whether its parameters remain consistent in different cultural contexts. Furthermore, we sought to establish standardized norms for interpreting ISCD scores, enhancing its cross-cultural applicability and interpretability.

## Material and methods

### Participants

The study involved 5,544 undergraduate and graduate students from Brazil, Portugal, and Spain. The Portuguese sample consisted of 222 students from the University of Algarve (72.97% female, M_age_ = 29.13, SD_age_ = 8.29), of whom 17 (7.6%) reported having a disability. The Spanish sample included 611 students from the University of Barcelona (76.92% female, M_age_ = 28.41, SD_age_ = 9.82), with 52 participants (8.51%) reporting a disability, and 676 students from the University of Seville (71.74% female, M_age_ = 26.01, SD_age_ = 5.62), of whom 20 (2.9%) reported having a disability. The Brazilian sample comprised 4,035 students from various public universities across all regions of the country (64.29% female, M_age_ = 31.24, SD_age_ = 9.17), with 251 participants (6.2%) reporting a disability. It is important to note that, due to Brazil’s large territorial extension and cultural diversity, the sample included students from public universities across all regions of the country. The distribution across regions was as follows: Central-West = 6.4%, Northeast = 12.9%, North = 5.9%, South = 7.6%, and Southeast = 67.9%. In the case of Spain, students from the University of Barcelona and the University of Seville were treated as separate samples due to linguistic characteristics, given that Catalan is the predominant language in Barcelona, whereas Spanish is the primary language in Seville, and therefore the ISCD was used in each sample in different languages. The inclusion criteria required participants to be 18 years of age or older, agree with the consent form, and to be enrolled in one of the participating universities. No exclusion criteria were applied.

### Measures

#### Sociodemographic information

We collected information regarding participants’ sex, age, enrolled course, university, university region (only for Brazil), and the presence or absence of disabilities.

#### Conception of disability

The ISCD is an instrument designed to identify general conceptions of disability across different cultural contexts; in other words, it is not intended to assess the specificities of any particular type of disability. It consists of 43 affirmative statements distributed across three conceptions: 17 statements related to the biological conception (which pertains to identifying disability based on biological or organic differences affecting the subject's ability or functionality, e.g., "Disability involves an organic malfunction that compromises human development"); 15 statements related to the social conception (which evaluates how social conditions impact disability, where functionality is only possible depending on whether the context excludes or includes, e.g., "Disability is defined according to the interpretation that each culture gives it"); and 11 statements related to the metaphysical conception (which measures how much the respondent associates disability with mystical meanings or links it to ideas of blessing or punishment, e.g., "The family of a person with a disability is prepared by God to receive them"). Each item is rated on a five-point Likert scale, where respondents select one option ranging from strongly disagree (1), disagree (2), neither agree nor disagree (3), agree (4), to strongly agree (5).

The ISCD was developed by Leite (2019) and was based on a previous 20-item instrument, the Scale on Conceptions of Disability (*Escala de Concepções de Deficiência*; Leite & Lacerda, 2018). The development of the ISCD was motivated by the need to expand the item set of the original SCD and to generate a measure capable of capturing conceptions of disability across diverse cultural contexts. To achieve this, a team of national and international researchers was assembled. The full set of procedures that guided the transition from the SCD to the ISCD is detailed in Leite et al. (2021).

These procedures included qualitative content validation conducted by expert judges from Brazil and other countries, as well as the examination of principal components through exploratory factor analysis using data collected from a Brazilian sample. Subsequently, four language versions of the instrument were developed: Brazilian Portuguese, European Portuguese, Spanish (Spain), and Catalan (Spain). The translation and back-translation processes were carried out by native speakers of each language, all of whom participated as researchers in the study coordinated by the instrument’s author (Leite et al., 2021).

### Procedures

We administered the ISCD online using Google Forms, part of the Google Workspace suite, with invitations sent to students through the institutional websites of the participating universities. Prior to accessing the ISCD, the first page of the form included the Informed Consent Form along with a contact email for inquiries, in accordance with ethical guidelines. After providing consent, students answered sociodemographic questions before proceeding to the ISCD scale. The formulary was configurated to require responses to all the items, therefore, we did not have any missing responses.

It is important to note that we conducted this research in compliance with the ethical standards outlined by the Declaration of Helsinki and the Brazilian National Health Council's Resolution No. 510 of April 7, 2016. The study was approved by the ethics committee under protocol No. 13476713.5.0000.5398 – CAAE/Plataforma Brasil, with a Certificate of Presentation for Ethical Consideration.

### Data analysis

Based on the previously established structure of the ISCD with a Brazilian sample (Leite et al., 2021), we tested a confirmatory factor model with three factors (social, metaphysical, and biological) for each locality in an attempt to replicate the reported structure. The confirmatory factor analyses were conducted using the weighted least squares mean and variance adjusted (WLSMV) estimator, which is robust against normality deviations. The adequacy of the factor structure was evaluated using the following fit indices and cutoffs: values of 0.95 or higher on the comparative fit index (CFI) and Tucker–Lewis index (TLI) indicate a good fit and values of 0.90 or higher are considered adequate; values below 0.05 on the root mean square error of approximation (RMSEA) represent a good fit while values below 0.10 are deemed adequate (Hu & Bentler, 1999; MacCallum et al., 1996; McDonald & Ho, 2002).

We tested the measurement invariance of the ISCD across localities using a multigroup confirmatory factor analysis (MGCFA). Following the recommendations of Wu and Estabrook (2016) for categorical data, we tested three models: the configural model (which constrains the number and composition of factors to be equal across groups), the proposition 4 model (which constrains thresholds to be equal across groups), and the proposition 7 model (which constrains both thresholds and factor loadings to be equal across groups). All models used delta parametrization and the WLSMV estimator. Measurement invariance was confirmed if changes in the fit indices between progressively restricted models met the following criteria: ∆χ^2^/∆df < 5; ∆RMSEA < 0.015; ∆CFI < 0.010 (Chen, 2007; Cheung & Rensvold, 2002).

We compared the mean ISCD scores across localities for the social, metaphysical, and biological dimensions using ANOVA with a Tukey post-hoc test to identify the source significant differences. Effect sizes were estimated using partial eta squared (η^2^p). Effect sizes of the post-hoc comparisons were estimated using Cohen’s d. A comparison was considered significant if *p* < 0.05. Additionally, we computed normative information for each ISCD score based on percentiles using the combined dataset (i.e., the pooled data from all localities). However, due to the disparity in sample sizes—particularly with Brazil having over 4,000 participants compared to fewer than 700 in the other localities—we randomly selected 676 participants from the Brazilian sample (a number equal to the largest international subsample, from Seville) to avoid over-representation of the Brazilian data in the normative calculations. Ensuring equal group contribution to the normative distribution is critical to avoid interpretative bias, as pooled norms are otherwise anchored in the score distribution of the largest cultural group. The subsampling procedure therefore protects the validity and fairness of cross-cultural comparisons. We then established four interpretative categories: disagreement, tendency towards disagreement, tendency towards agreement, and agreement, corresponding to the 1st–25th, 26th–50th, 51st–75th, and 76th–100th percentiles, respectively.

All the analysis were performed in R (version 4.4.1). The confirmatory factor analyses were conducted using the lavaan (Rosseel, 2012) package. The MGCFA was performed with the semTools (Jorgensen et al., 2022) and lavaan (Rosseel, 2012) packages. Mean comparisons were carried out using the jmv (Selker et al., 2022) package, while normative information was computed with the base R faction ‘quantile’.

## Results

We first conducted a CFA for each locality to assess the fit of the three-factor structure of the ISCD. The fit indices for each tested model are presented in Table 1.

**Table 1:** Fit indices of the ISCD confirmatory factor models by locality

Locality	χ^2^	df	CFI	TLI	RMSEA [95% CI]
Barcelona	2,795.83*	857.00	0.95	0.94	0.061 [0.058—0.063]
Sevilla	3,011.06*	857.00	0.92	0.92	0.061 [0.059—0.063]
Portugal	1,588.80*	857.00	0.94	0.94	0.062 [0.057—0.067]
Brazil	12,393.69*	857.00	0.93	0.92	0.058 [0.057—0.059]

^*^p < 0.05, *df* degrees of freedom, *CFI* Comparative Fit Index, *TLI* Tucker-Lewis Index, *RMSEA* Root Mean Square Error of Approximation, *CI* Confidence Interval

The fit indices indicated that the three-factor structure of the ISCD was adequate across all localities. The factor loadings, factor correlations, and internal consistency estimates (Cronbach’s alpha and McDonald’s omega) for all samples are presented in the Supplementary Material. After confirming the ISCD structure, we proceeded to test its measurement invariance between the different localities. Table 2 presents the fit indices of the configural, threshold (proposition 4) and threshold + loadings (proposition 7) invariance models.

**Table 2:** Fit indices of the multi-group confirmatory factor analysis of ISCD across localities

	χ^2^	df	χ^2^/df (∆)	CFI (∆)	TLI (∆)	RMSEA [95% CI]	∆ RMSEA
baseline	16,621.68*	3428	4.85 (-)	0.938 (-)	0.935 (-)	0.053 [0.052—0.054]	-
prop4	17,167.22*	3686	4.66 (2.11)	0.937 (0.001)	0.938 (0.003)	0.051 [0.051—0.052]	0.002
prop7	17,076.61*	3806	4.47 (0.75)	0.938 (0.001)	0.941 (0.003)	0.050 [0.049—0.051]	0.001

^*^p < 0.05, *df* degrees of freedom, *CFI* Comparative Fit Index, *TLI* Tucker-Lewis Index, *RMSEA* Root Mean Square Error of Approximation, *CI* Confidence Interval, ∆ = difference

The multigroup confirmatory factor analysis supported the full measurement invariance of the ISCD across the tested cultures. Given that the scale's parametrization operates equivalently across the different localities, we compared the means of the different localities on the ISCD scores, for which the results are presented in Table 3.

**Table 3:** Mean comparison on the ISCD scores between localities

	Locality	N	M	SD	F	*p*	η^2^p	Tukey
Social	1 = Barcelona	611	47.07	11.47	32.76	< 0.001	0.017	3 < 1;2;4
	2 = Brazil	4035	47.12	12.56				
	3 = Portugal	222	38.83	11.69				
	4 = Seville	676	47.45	11.54				
Metaphysical	1 = Barcelona	611	14.08	7.36	43.13	< 0.001	0.022	1;4 < 2;3
	2 = Brazil	4035	17.23	8.47				
	3 = Portugal	222	16.49	8.19				
	4 = Seville	676	14.44	6.73				
Biological	1 = Barcelona	611	50.38	12.46	4.86	0.002	0.003	2,4 < 3
	2 = Brazil	4035	49.83	13.22				
	3 = Portugal	222	53.03	13.62				
	4 = Seville	676	49.42	11.81				

Portugal exhibited significantly lower scores in the social deficiency conception compared to the other localities (d_Barcelona x Portugal_ = 0.67; d_Brasil x Portugal_ = 0.67; d_Seville x Portugal_ = 0.70), while no significant differences were observed between the other countries. Barcelona and Seville had significantly lower scores on the metaphysical conception compared to Brazil (d_Barcelona x Brazil_ = 0.39; d_Seville x Brazil_ = 0.34) and Portugal (d_Barcelona x Portugal_ = 0.30; d_Seville x Portugal_ = 0.25). In terms of the biological conception, Portugal had significantly higher scores compared to Brazil (d = 0.25) and Seville (d = 0.28). Even though the highlighted results were statistically significant, only the differences in the social conception of disability showed medium effect sizes, whereas the differences in the other conceptions exhibited small effect sizes.

With the goal of enhancing the practical application of the ISCD, we also calculated the normative data for the scales using the combined sample. The decision to establish a single aggregated normative score across all samples is supported by the demonstrated measurement invariance of the instrument across the three countries. Measurement invariance ensures that any observed differences reflect true variation in the underlying construct, rather than differences in how the measure functions across groups. Creating separate normative scores for each country would compromise comparability, as the same score value could be interpreted as “low” in one context and “high” in another, thereby removing the shared frame of reference needed for cross-cultural comparison. This normative information is expected to serve as a valuable reference for future users of the ISCD, providing guidance on interpreting scores within different cultural contexts. The normative information is presented in Table 4. We divided the scores into four interpretative categories: disagreement, tendency towards disagreement, tendency towards agreement, and agreement. The score range of each of these categories provide normative reference points that help interpret individual or group scores on the ISCD. Users of the scale can utilize these cut-offs to categorize responses and better understand the tendencies in the endorsement of different deficiency conceptions.

**Table 4:** Normative information for the ISCD scores

Deficiency conceptions	Disagreement (1–25%)	Tendency towards disagreement (26–50%)	Tendency towards agreement (51–75%)	Agreement (76–100%)
Social	15 to 38	39 to 47	48 to 55	56 to 75
Metaphysical	11	12	13 to 16	17 to 55
Biological	17 to 42	43 to 51	52 to 59	60 to 85

## Discussion

Therefore, the aim of this study was to investigate the validity of the Intercultural Scale of Conceptions of Disability in countries beyond Brazil (where the scale was originally developed; Leite et al., 2020), specifically Portugal and Spain. In addition to examining the internal structure of the measure, we tested its measurement invariance across countries and provided normative data for interpreting the scale scores, enhancing its applicability and interpretability. Overall, our results suggest that the internal structure is robust across all assessed countries, and measurement invariance is upheld. This indicates that the instrument's parameters are equivalent across countries, allowing for direct and accurate comparisons of scores between different regions.

Our results indicated that the three-factor structure of the ISCD demonstrated a good fit across all locations. This suggests that conceptions of disability can be understood as a multidimensional construct consisting of biological (which pertains to understanding disability as an organic difference, where the individual's perceived incapacity is attributed to biological differences), social (which recognizes that individuals may present organic or behavioral differences, but these alone do not constitute disability, rather, disability is determined by the sociocultural context in which the individual is embedded), and metaphysical (which measures the extent to which respondents associate disability with religious or mystical meanings that transcend human understanding). These findings are consistent with the validation study conducted on the Brazilian version of the ISCD (Leite et al., 2021). In addition to maintaining the same factor structure across countries, the ISCD also demonstrated comparable thresholds for response categories and similar factor loadings across different populations, as shown by our measurement invariance results (Gregorich, 2006; Wu & Estabrook, 2016). These findings ensure that the scale reliably and comparably assesses conceptions of disability across cultures, providing robust evidence of the ISCD’s cross-cultural validity.

The mean comparison results revealed significant differences in conceptions of disability across the assessed locations. The largest difference was found in the metaphysical conception (η^2^p = 0.022), with Spain scoring higher than both Brazil and Portugal. Additionally, Portugal exhibited significantly lower levels of the social conception of disability compared to the other countries. The biological conception also showed significant differences across countries, though with a small effect size (η^2^p = 0.003). Portugal had significantly higher scores compared to Brazil and Seville. These differences suggest that, although these countries are signatories of common international agreements, such as the Convention on the Rights of Persons with Disabilities, their specific characteristics can influence how disability is interpreted, as highlighted in previous studies (Borges et al., 2022; Gràcia et al., 2022; Leite et al., 2023).

Although our results provide valuable insights regarding the ISCD's functioning and applicability in different contexts, they must be interpreted in light of the study's main limitations. First, while we had a very large Brazilian sample, the samples from the other locations were not as representative, which may reduce the variability in their responses and potentially limit the generalizability of our findings. This limitation is especially notable in the case of Portugal, where the sample size was the smallest and drawn exclusively from a single university. Second, the study only included university samples, which could further impair the generalizability of the results to other populations. Third, although our study provides strong evidence of the ISCD’s validity based on its internal structure across countries, it does not provide evidence of validity based on external measures or behavioral/criterion-related outcomes. We recommend that future studies address these issues by including larger and more representative samples from other countries, as well as expanding the use and validation of the ISCD in different localities and cultures.

Studying conceptions related to a given construct is important because meanings are constructed and shared through interactive dynamics and are continually reshaped through dialogical processes grounded in social experience (Vygotsky, 2010). These experiences may be direct or mediated by the internet, media, books, and magazines, among other sources. The importance of understanding and assessing conceptions of disability is particularly evident, as it allows us to identify how different social groups position themselves in relation to the various perspectives circulating in society; perspectives that shape how we act, label, and behave toward individuals with disabilities (Leite et al., 2023a; Leite et al., 2023b; Nepomuceno et al., 2020). In short, this involves understanding which predominant tendency exists within the group under analysis when it comes to interpreting disability, namely, whether respondents align with conceptions grounded in a social model (understood as historically and culturally situated), a biological model (focused on the individual and aligned with clinical-medical explanations), or a metaphysical model (which attributes causes to religious or spiritual interpretations). Such understanding provides elements for critically analyzing respondents’ positioning and its implications for the field under study.

## Conclusion

Developing research instruments aimed at examining concepts related to disability that can be applied to future professionals is crucial, as the discomfort caused by individuals perceived as abnormal—those who diverge from societal expectations—can hinder the provision of developmental opportunities for people with disabilities (Barroco & Leite, 2021). This can intensify a normalized process of exclusion for this group, as the conditions in place are often not adapted to accommodate different bodies, leading to either their exclusion or the offering of limited opportunities for social participation.

The contributions of this study focus on presenting the psychometric properties of a scale capable of demonstrating how different social groups respond to three frameworks for interpreting the analytical category of disability. The results can be analyzed in isolation for specific samples or in a comparative manner (intragroup—pre- and post-test—or intergroup comparisons). Based on the data presented in this study, it is possible to design and implement micro and macro level studies and interventions aimed at rethinking how society portrays disability and promoting actions that encourage more inclusive practices. This can be achieved through the analysis of ISCD results obtained from diverse and distinct social groups. The findings also reinforce conclusions from previous research conducted in various educational contexts, including Brazil (Facin et al., 2024; Leite et al., 2023), Spain (Gràcia et al., 2022; Pereira-Leite et al., 2023), and Portugal (Borges et al., 2022), allowing for an examination of how university students position themselves in relation to the conceptions captured by the scale. In other words, this understanding has the potential to inform the creation of targeted interventions, whether focused on specific groups or implemented more broadly within educational institutions, workplaces, or other social contexts. In this way, it is expected that we can advance the understanding of perceptions and conceptions related to disability, contributing to the development of more effective actions that are sensitive to diverse realities and to the individuals who experience them.

In addition, the results offer a valuable tool for researchers and professionals who wish to assess the impact of interventions and programs aimed at inclusion, that is, the greater participation of people with disabilities in different social spheres, enabling adjustments in the context based on concrete evidence. In this way, the study not only expands theoretical knowledge about social responses to disability, but also provides practical measures to promote a more just, accessible, and welcoming society, in which structural, cognitive, sensory, and/or behavioral differences are recognized and valued as an integral part of the human condition.

## Supplementary Information

Supplementary Material 1.

## Data Availability

This study was not preregistered. All data and codes have been made publicly available at the Institutional Repository Unesp and can be accessed at https://hdl.handle.net/11449/257821.

## Abbreviations

ADS: Attitudes to Disability Scale
CATCH: Chedoke McMaster Attitudes Toward Children with Handicaps
CFA: Confirmatory factor analysis
CFI: Comparative fit index
DSW: Disability Social Work Scale
EAD: Escala de Actitudes hacia la Discapacidad
ECD: Escala de Concepções de Deficiência
ISCD: Intercultural Scale on Conceptions of Disability
MGCFA: Multigroup confirmatory factor analysis
RMSEA: Root mean square error of approximation
TLI: Tucker–Lewis index
WLSMV: Weighted least squares mean and variance adjusted

## Footnotes

1 For more detailed information, refer to the United Nations Disability Inclusion Strategy available at the UN website https://www.un.org/en/content/disabilitystrategy/.
