# Supplementary material for: Intercultural Scale on Conceptions of Disability: a cross-cultural validation
Source: Psicol Reflex Crit. 2026 Mar 14;39:13. doi: 10.1186/s41155-026-00379-0 (PMC13038782; doi:10.1186/s41155-026-00379-0)
Supplement: Supplementary file 1 — Supplementary Material 1. [file 41155_2026_379_MOESM1_ESM.docx]

**Supplementary Material**

**Intercultural Scale of Conceptions of Disability: a cross-cultural validation**

**Supplementary Table 1.**

CFA Barcelona – Structure, factor loadings, and internal consistency

|  | Social | Metaphysical | Biological |
| --- | --- | --- | --- |
| EICD1 | 0.49 |  |  |
| EICD2 |  |  | 0.54 |
| EICD3 | 0.42 |  |  |
| EICD4 | 0.61 |  |  |
| EICD5 |  | 0.86 |  |
| EICD6 |  |  | 0.56 |
| EICD7 | 0.56 |  |  |
| EICD8 |  | 0.94 |  |
| EICD9 |  | 0.94 |  |
| EICD10 |  | 0.98 |  |
| EICD11 |  | 0.95 |  |
| EICD12 |  |  | 0.63 |
| EICD13 | 0.70 |  |  |
| EICD14 |  | 0.94 |  |
| EICD15 |  |  | 0.43 |
| EICD16 |  |  | 0.61 |
| EICD17 |  | 0.93 |  |
| EICD18 |  |  | 0.71 |
| EICD19 |  |  | 0.61 |
| EICD20 |  | 0.96 |  |
| EICD21 |  |  | 0.73 |
| EICD22 |  |  | 0.70 |
| EICD23 |  |  | 0.53 |
| EICD24 | 0.70 |  |  |
| EICD25 | 0.58 |  |  |
| EICD26 |  |  | 0.74 |
| EICD27 | 0.79 |  |  |
| EICD28 | 0.76 |  |  |
| EICD29 |  |  | 0.73 |
| EICD30 | 0.57 |  |  |
| EICD31 | 0.72 |  |  |
| EICD32 |  |  | 0.75 |
| EICD33 |  |  | 0.79 |
| EICD34 |  |  | 0.61 |
| EICD35 |  | 0.96 |  |
| EICD36 | 0.62 |  |  |
| EICD37 |  | 0.88 |  |
| EICD38 |  | 0.90 |  |
| EICD39 |  |  | 0.80 |
| EICD40 | 0.79 |  |  |
| EICD41 | 0.76 |  |  |
| EICD42 | 0.63 |  |  |
| EICD43 |  |  | 0.63 |
| α | 0.89 | 0.96 | 0.91 |
| ω | 0.89 | 0.96 | 0.91 |

The correlations between the factors are presented below. Social and Metaphysical: r = 0.069 (p = 0.147). Social and Biological: r = –0.073 (p = 0.070). Metaphysical and Biological: r = 0.143 (p = 0.012).

**Supplementary Table 2.**

CFA Seville – Structure, factor loadings, and internal consistency

|  | Social | Metaphysical | Biological |
| --- | --- | --- | --- |
| EICD1 | 0.53 |  |  |
| EICD2 |  |  | 0.57 |
| EICD3 | 0.48 |  |  |
| EICD4 | 0.60 |  |  |
| EICD5 |  | 0.87 |  |
| EICD6 |  |  | 0.63 |
| EICD7 | 0.55 |  |  |
| EICD8 |  | 0.91 |  |
| EICD9 |  | 0.91 |  |
| EICD10 |  | 0.96 |  |
| EICD11 |  | 0.89 |  |
| EICD12 |  |  | 0.67 |
| EICD13 | 0.75 |  |  |
| EICD14 |  | 0.87 |  |
| EICD15 |  |  | 0.24 |
| EICD16 |  |  | 0.70 |
| EICD17 |  | 0.89 |  |
| EICD18 |  |  | 0.73 |
| EICD19 |  |  | 0.66 |
| EICD20 |  | 0.89 |  |
| EICD21 |  |  | 0.68 |
| EICD22 |  |  | 0.63 |
| EICD23 |  |  | 0.56 |
| EICD24 | 0.63 |  |  |
| EICD25 | 0.65 |  |  |
| EICD26 |  |  | 0.64 |
| EICD27 | 0.79 |  |  |
| EICD28 | 0.71 |  |  |
| EICD29 |  |  | 0.72 |
| EICD30 | 0.60 |  |  |
| EICD31 | 0.78 |  |  |
| EICD32 |  |  | 0.76 |
| EICD33 |  |  | 0.77 |
| EICD34 |  |  | 0.53 |
| EICD35 |  | 0.92 |  |
| EICD36 | 0.72 |  |  |
| EICD37 |  | 0.88 |  |
| EICD38 |  | 0.84 |  |
| EICD39 |  |  | 0.77 |
| EICD40 | 0.82 |  |  |
| EICD41 | 0.75 |  |  |
| EICD42 | 0.59 |  |  |
| EICD43 |  |  | 0.54 |
| α | 0.90 | 0.94 | 0.90 |
| ω | 0.90 | 0.94 | 0.90 |

The correlations between the factors are presented below. Social and Metaphysical: r = –0.038 (p = 0.440). Social and Biological: r = 0.099 (p = 0.011). Metaphysical and Biological: r = 0.255 (p < 0.001).

**Supplementary Table 3.**

CFA Portugal – Structure, factor loadings, and internal consistency

|  | Social | Metaphysical | Biological |
| --- | --- | --- | --- |
| EICD1 | 0.62 |  |  |
| EICD2 |  |  | 0.55 |
| EICD3 | 0.33 |  |  |
| EICD4 | 0.69 |  |  |
| EICD5 |  | 0.85 |  |
| EICD6 |  |  | 0.66 |
| EICD7 | 0.61 |  |  |
| EICD8 |  | 0.91 |  |
| EICD9 |  | 0.92 |  |
| EICD10 |  | 0.93 |  |
| EICD11 |  | 0.88 |  |
| EICD12 |  |  | 0.72 |
| EICD13 | 0.68 |  |  |
| EICD14 |  | 0.89 |  |
| EICD15 |  |  | 0.54 |
| EICD16 |  |  | 0.69 |
| EICD17 |  | 0.90 |  |
| EICD18 |  |  | 0.83 |
| EICD19 |  |  | 0.63 |
| EICD20 |  | 0.84 |  |
| EICD21 |  |  | 0.75 |
| EICD22 |  |  | 0.70 |
| EICD23 |  |  | 0.61 |
| EICD24 | 0.68 |  |  |
| EICD25 | 0.57 |  |  |
| EICD26 |  |  | 0.81 |
| EICD27 | 0.80 |  |  |
| EICD28 | 0.79 |  |  |
| EICD29 | 0.64 |  | 0.83 |
| EICD30 | 0.78 |  |  |
| EICD31 | 0.73 |  |  |
| EICD32 |  |  | 0.79 |
| EICD33 |  |  | 0.75 |
| EICD34 |  |  | 0.72 |
| EICD35 |  | 0.92 |  |
| EICD36 | 0.73 |  |  |
| EICD37 |  | 0.83 |  |
| EICD38 |  | 0.85 |  |
| EICD39 |  |  | 0.79 |
| EICD40 | 0.79 |  |  |
| EICD41 | 0.81 |  |  |
| EICD42 | 0.66 |  |  |
| EICD43 |  |  | 0.73 |
| α | 0.90 | 0.94 | 0.93 |
| ω | 0.90 | 0.94 | 0.93 |

The correlations between the factors are presented below. Social and Metaphysical: r = 0.334 (p < 0.001). Social and Biological: r = 0.089 (p = 0.089). Metaphysical and Biological: r = –0.062 (p = 0.306).

**Supplementary Table 4.**

CFA Brazil – Structure, factor loadings, and internal consistency

|  | Social | Metaphysical | Biological |
| --- | --- | --- | --- |
| EICD1 | 0.55 |  |  |
| EICD2 |  |  | 0.52 |
| EICD3 | 0.49 |  |  |
| EICD4 | 0.70 |  |  |
| EICD5 |  | 0.82 |  |
| EICD6 |  |  | 0.64 |
| EICD7 | 0.68 |  |  |
| EICD8 |  | 0.91 |  |
| EICD9 |  | 0.92 |  |
| EICD10 |  | 0.90 |  |
| EICD11 |  | 0.86 |  |
| EICD12 |  |  | 0.57 |
| EICD13 | 0.64 |  |  |
| EICD14 |  | 0.85 |  |
| EICD15 |  |  | 0.45 |
| EICD16 |  |  | 0.68 |
| EICD17 |  | 0.84 |  |
| EICD18 |  |  | 0.69 |
| EICD19 |  |  | 0.64 |
| EICD20 |  | 0.81 |  |
| EICD21 |  |  | 0.67 |
| EICD22 |  |  | 0.69 |
| EICD23 |  |  | 0.51 |
| EICD24 | 0.74 |  |  |
| EICD25 | 0.60 |  |  |
| EICD26 |  |  | 0.69 |
| EICD27 | 0.77 |  |  |
| EICD28 | 0.76 |  |  |
| EICD29 |  |  | 0.71 |
| EICD30 | 0.50 |  |  |
| EICD31 | 0.71 |  |  |
| EICD32 |  |  | 0.71 |
| EICD33 |  |  | 0.75 |
| EICD34 |  |  | 0.61 |
| EICD35 |  | 0.91 |  |
| EICD36 | 0.70 |  |  |
| EICD37 |  | 0.85 |  |
| EICD38 |  | 0.76 |  |
| EICD39 |  |  | 0.74 |
| EICD40 | 0.84 |  |  |
| EICD41 | 0.78 |  |  |
| EICD42 | 0.57 |  |  |
| EICD43 |  |  | 0.59 |
| α | 0.90 | 0.92 | 0.90 |
| ω | 0.90 | 0.93 | 0.90 |

The correlations between the factors are presented below. Social and Metaphysical: r = –0.013 (p = 0.461). Social and Biological: r = –0.213 (p < 0.001). Metaphysical and Biological: r = 0.276 (p < 0.001).
